# Development and evaluation of oral Cancer quality-of-life questionnaire (QOL-OC)

**DOI:** 10.1186/s12885-018-4378-6

**Published:** 2018-05-03

**Authors:** Min Nie, Chang Liu, Yi-Chen Pan, Chen-Xi Jiang, Bao-Ru Li, Xi-Jie Yu, Xin-Yu Wu, Shu-Ning Zheng

**Affiliations:** The State Key Laboratory Breeding Base of Basic Science of Stomatology, Hubei Province & Key Laboratory of Oral Biomedicine (Wuhan University), Ministry of Education, School and Hospital of Stomatology, Wuhan University, Luoyu Road 237, Wuhan, 430079 Hubei China

**Keywords:** Oral Cancer quality-of-life questionnaire, QOL-OC, Chinese oral cancer, Chinese version

## Abstract

**Background:**

In this study scales and items for the Oral Cancer Quality-of-life Questionnaire (QOL-OC) were designed and the instrument was evaluated.

**Methods:**

The QOL-OC was developed and modified using the international definition of quality of life (QOL) promulgated by the European Organization for Research and Treatment of Cancer (EORTC) and analysis of the precedent measuring instruments. The contents of each item were determined in the context of the specific characteristics of oral cancer. Two hundred thirteen oral cancer patients were asked to complete both the EORTC core quality of life questionnaire (EORTC QLC-C30) and the QOL-OC. Data collected was used to conduct factor analysis, test-retest reliability, internal consistency, and construct validity.

**Results:**

Questionnaire compliance was relatively high. Fourteen of the 213 subjects accepted the same tests after 24 to 48 h demonstrating a high test-retest reliability for all five scales. Overall internal consistency surpasses 0.8. The outcome of the factor analysis coincides substantially with our theoretical conception. Each item shows a higher correlation coefficient within its own scale than the others which indicates high construct validity.

**Conclusions:**

QOL-OC demonstrates fairly good statistical reliability, validity, and feasibility. However, further tests and modification are needed to ensure its applicability to the quality-of-life assessment of Chinese oral cancer patients.

**Electronic supplementary material:**

The online version of this article (10.1186/s12885-018-4378-6) contains supplementary material, which is available to authorized users.

## Background

Oral cancer includes cancers of the oral cavity and adjacent anatomical sites. The incidence rate of oral cancer ranks sixth among systemic cancers and first among head and neck cancers [1]. Occurrence of oral cancer continues to increase and developing countries experience higher rates of morbidity and mortality from oral cancer than do developed countries [2]. Due to the specific anatomical sites and structures that characterize oral cancer, both the disease process and its treatment may greatly impair the body image and practical functions of patients in even the most essential life tasks such as breathing, speaking, swallowing, and eating [3, 4]. During the past six decades, both the mortality and 5-year survival rates among oral cancer patients basically remain unchanged [5, 6]. For these reasons, the improvement of quality of life has gained the attention of the medical community [7]. Furthermore, quality of life assessments have been providing evidence that is critical to both the assessment of patients’ living states and the formulation of clinical strategies [8–10].

Standardized measuring instruments are used to assess quality of life [11, 12]. Widely used QOL instruments include the European Organization for Research and Treatment of Cancer (EORTC) QLQs [13, 14], the Functional Assessment of Cancer Therapy (FACT) scale [15], and the University of Washington Head and Neck Measure (UW-QOL) and the Functional Living Index-Cancer (FLIC) [16], and so on. Neither the questionnaires mentioned above nor other generic measures like the Medical Outcomes Short Form 36 (SF-36) [17] are specifically tailored to the problems oral cancer patients experience [18, 19]. Assessment of oral cancer has been borrowing scales of head and neck cancer, such as EORTC H&N35, FACT-H&N, and so on. In fact, patients with oral cancer are more likely to suffer from more significant dental problems, more limited mouth opening, more severe swallowing, chewing, speech and saliva secretion problems than other head and neck cancers (such as laryngeal cancer, pharyngeal cancer, and so on) [20–26]. Different anatomical sites and its functions are bound to be the limitation of life questionnaire with head and neck cancer [13, 21, 27–29]. Furthermore, there is dearth of specific measures designed for use within the context of Chinese culture. Therefore, there is a need for a questionnaire designed to assess the quality of life of Chinese oral cancer patients.

## Methods

### Development of the QOL-OC

The questionnaire consists of a general module and a specific module. The EORTC QLQ-C30 and its core measures for cancer patients were directly adopted as the general module. The QLC-C30 contains a total of 30 questions covering the basic aspects of health-related quality of life. Its five function scales are physical functioning, role functioning, cognitive functioning, emotional functioning, and social functioning; the remainder of the questionnaire consists of symptom scales. The questionnaire is used in the treatment of patients suffering from all categories of cancer [28]. The Chinese version of the C30 enjoys relatively high reliability, validity, and feasibility as well as wide recognition in China [30].

The oral-cancer-specific module is based on the guidelines of the EORTC Quality of Life Group [31–34] as well as the definition of QOL by the World Health Organization (WHO) as an individual’s “perception of their position in life in the context of culture and value system in their life and in relation to their goals, expectations, standards and concerns” [35].

Drawing on relevant papers collected from databases like PubMed and SSIC and opinions from patients and experts, we amalgamated, deleted, rephrased, and added some of the current questionnaire items. These decisions were based mainly on the current version of the EORTC H&N35; the overall purpose of this work was to detect the problems in currently available measures and revise accordingly. Considering the limitations of the instrument, an open-ended question was added to supplement it.

The QOL-OC consists of 29 items among 15 scales. The first 26 of these 29 items are scored on a four-point Likert scale: meiyou (not at all), youyidian (a little), bijiaoduo (quite a bit), feichangduo (very much). Items 27 and 28 item are yes/no questions; yes responses are scored with 1 point and no responses are scored with 0. The last item, number 29, is an open-ended question which does not contribute to the numerical score and is only used for material collection. The division of scales and the scoring procedure are shown in Table 3.

### Data collection

Each subject (more than 18) signed a formal Informed Consent statement, and the entire study was approved by the Medical Ethics Committee of Wuhan University. Data collection began in June 2014 and ended in March 2015. Patients were selected from Wuhan University School of Stomatology, Wuhan University Zhongnan Hospital, Changzhou Stomatology Hospital, and Shenzhen No. 2 People’s Hospital. The majority of the patients were interviewed telephonically and the rest were interviewed face-to-face. Some of the patients was interviewed again 24 to 48 h later solely to establish test-retest reliability. Inclusion and exclusion criteria are as follows:

Inclusion criteria:

1. Pathologically diagnosed with oral cancer;
2. 18 or older;
3. Estimated survival time longer than 3 months;
4. Aware of the diagnosis;
5. Able to understand and answer the questionnaire on their own or with the explanation of investigators;
6. Involved voluntarily.

Exclusion criteria:

1. With mental illness, disturbances in conscious mental processes, or communication barriers;
2. Refusing to be involved in this study or asking to quit during the study.

### Data analysis

Statistical analysis was conducted using SPSS 19.0. Internal consistency was assessed using Cronbach’s alpha, and test-retest ability was assessed using the Spearman rank-order correlation coefficient. Factor analysis (principal axis factor analysis), along with parallel analysis were conducted to judge the division of scales, and convergent and discriminant construct validity were evaluated using the Spearman rank-order correlation coefficient.

## Results

### Development of questionnaire

The EORTC-H&N35 [13] and other commonly used head and neck measures such as FACT-H&N [36] and UW-QOL [37] were used to develop the QOL-OC questionnaire. QOL-OC used EORTC-H&N35 as it prototype and according to the pilot study, specific questions intimately related to the oral cavity region such as shoulder and neck function, saliva secretion were added to the instrument and less significantly related items from the sources were eliminated. This resulted in a questionnaire containing 29 questions, see Additional file 1.

Based on the information gleaned from the discussion of the panel and patients, several questions were fine-tuned to be more acceptable and easily understood by patients. It is worth noting that, drawing from the Chinese version of the EOTRC QLQ-C30, we used a four point scale: ‘meiyou (not at all)’, ‘youyidian (a little)’, ‘xiangdangduo (quite a lot)’, and ‘feichangduo (very much)’. However, during the discussion patients reported difficulty distinguishing between ‘xiangdangduo (quite a lot)’ and ‘feichangduo (very much)’ in Chinese. Therefore, we rephrased ‘xiangdangduo’ into ‘bijiaoduo (relatively much)’ in the actual interviews.

We analyzed data and results from the current publications and working papers concerning the appraisal of head and neck specific measuring instruments, and extracted several oral functions that were not covered in our prototype frequently complained by oral cancer patients such as shoulder and neck function [21, 38], mastication [37, 39]. We then added other oral problems that patients might confront such as oral ulcer, enjoyment of food, diet change and bleeding gingiva. Since problems with speech is much less prominent among oral cancer patients than others like laryngeal cancer patients [13, 40], speech item was removed and substituted by pronunciation which is more related to organs in oral cavity than throat. Sense of smell was also removed for similar reason that dysosmia occurs more on nasopharynx cancer patients [13, 41]. The two questions concerning sexuality were combined considering the high internal consistency [13, 19, 42].

### Patient characteristics

A total of 213 patients were included. Significantly more subjects were males and the study included patients with a broad range of cancer sites (Table 1).

**Table 1:** Demographic information of patients included

Characteristics	No. of patients	%
Gender		
Male	139	65.3
Female	74	34.7
Age		
Mean (SD)	53.84(10.48)	
Range	18–86	
Marital status		
Married	197	92.5
Unmarried	8	3.8
Widowed	5	2.3
Divorced	2	.9
Education level		
Junior high	73	34.3
Senior high	60	28.2
Undergraduate or higher	41	19.2
Primary school or lower	30	14.1
Information missed	9	4.2
Cancer site		
Tongue	51	23.9
Buccal mucosa	29	13.6
Gingiva	25	11.7
Salivary glands	25	11.7
Mouth floor	23	10.8
Palate	23	10.8
Multi-sites	10	4.7
Lips	10	4.7
Maxilla/Mandible	8	3.8
Oropharynx	5	2.3
branchial fissure	1	.5
Temple	1	.5
Maxillary sinus	1	.5
Submandibular	1	.5
Pathological type		
SCC	144	67.6
Mucoepidermoid carcinoma carcinoma	12	5.6
Adenoid cystic	11	5.2
Adenocarcinoma	6	2.8
Pleomorphic adenoma	4	1.9
Other	30	14.6
Ameloblastoma	1	.5
Malignant Melanoma	2	.9
Hodgkin lymphoma	1	.5
Non-Hodgkin lymphoma	2	.9
Myoepithelial carcinoma	3	1.4
Basal cell carcinoma	3	1.4
Mesenchymal sarcoma	1	.5
Plasma cell sarcoma	2	.9
Sarcomatoid carcinoma	1	.5
Mesenchymal carcinoma	2	.9
Epithelioid vascular endothelium	1	.5
Spindle cell sarcoma	1	.5
Fibroblastoma	1	.5
Acinic cell carcinoma	4	1.9
Adenogenous low-grade malignancy	3	1.4
Dentinogenic ghost cell tumour	1	.5
Verrucous carcinoma	1	.5
Information missed	6	2.8
Treatment		
Surgery	200	93.9
Surgery/radiotherapy	8	3.8
Radiotherapy	2	.9
Surgery/radiotherapy/chemotherapy	1	.5
Surgery/chemotherapy	1	.5
Palliative treatment	1	.5

### Compliance

During the data collection period, a total of 282 patients were called or interviewed. Fifty-five directly refused and 10 failed to complete all the questions. Therefore, 213 effective questionnaires for each measuring instrument were obtained indicating a response rate of 75.5%. Subjects spent 10.4 min on average completing the questionnaire.

### Descriptive statistics

The patient responses to C30 described a general quality of life that was slightly low (score for global health status was 74.53 ± 19.92). Though physical functioning (90.58 ± 14.86), emotional functioning (93.19 ± 11.21), and cognitive functioning (91.47 ± 11.85) were fairly good in the context of the function scales, role functioning (88.58 ± 19.49) and social functioning (86.31 ± 21.14) lagged behind. In the context of the symptom scales, patients responses reflected prominent economic problems most often, followed by sleeping problems (14.40 ± 25.11), fatigue (13.67 ± 16.85), and pain (10.80 ± 16.92).

All QOL-OC scales are symptom scales. Notable eating (13.42 ± 15.11) and saliva problems (22.85 ± 23.06) were reported most frequently, whereas problems with pain and discomfort (8.58 ± 11.95), sexuality (6.57 ± 20.19), oral ulcers (8.45 ± 17.48), and bleeding gingival (5.16 ± 17.11) were less frequently reported (mean < 10). Weight gain (27.70 ± 44.86) occurred more than weight loss (18.31 ± 38.77). The scores of these two questionnaires can be seen in Additional files 2 and 3.

### Factor analysis

**Fig. 1:**
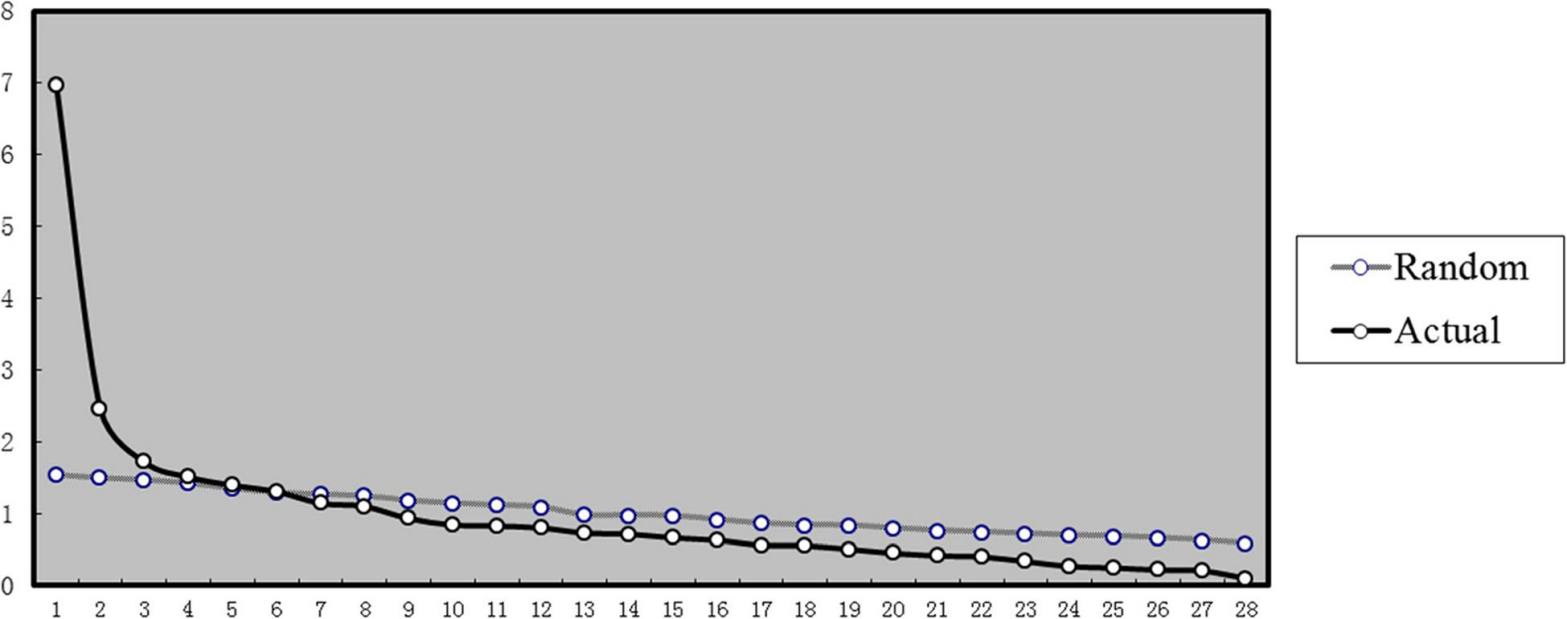
Scree plot of factor analysis along with parallel analysis. X-axis shows number of factors, Y-axis shows eigenvalues (eigenvalue greater than 1.0 Rule). Black line stands for actual data while grey line stands for random data

Factor analysis was performed to assess the division of scales. Parallel analysis was conducted to help decide the number of factors. Scree plot (Fig. 1) was drawn from actual eigenvalues obtained from principal axis factor analysis by varimax rotation and random eigenvalues got from parallel analysis.

According to the scree plot, 6 factors should be extracted. But considering the slightly low variances contribution (54.89%), 7 factors (58.97%) were selected instead [43] (Table 2). Factor 1 reflects problems of social contact. Factor 3 is mainly concerned with pain. Factor 4 is about problems with eating. Factor 5 involves problems with diet, weight change and sense of taste. Factor 7 reflects problems with saliva.

**Table 2:** Factor loadings of principal axis factor analysis

Factors	1	2	3	4	5	6	7
1 Pain in mouth			.585				
2 Pain in jaw			.574				
3 Pain in throat			.343		.394		
4 Discomfort in mouth		.583					
5 Eating fluid				.927			
6 Eating semifluid				.605			
7 Eating solid		.588					
8 Choked when eating						.502	
9 Teeth problem		.346					
10 Difficulty opening mouth		.529					
11 Dry mouth							.662
12 Sticky saliva							.533
13 Sense of taste					.371		
14 Appearance						.350	
15 Difficulty chatting	.831						
16 Social contact with family	.792						
17 Social contact with friends	.849						
18 Social contact in public	.819						
19 Sexuality						.441	
20 Oral ulcer		.241					
21 Trouble enjoying food					.422		
22 Diet change		.403			.394		
23 Pronunciation	.628						
24 Shoulder & neck function						.359	
25 Bleeding gingiva		.094					
26 Painkillers			.530				
27 Weight loss					.513		
28 Weight gain					−.379		

Only the maximum loadings of each item were shown in the table

In both statistically and clinically justified sense, the final scale division was established as shown in Table 3.

**Table 3:** Scoring Methods and Test-retest ability of QOL-OC

Scale	Scales	Items	Score Range	Min	Max	Rough Score	Standardized Score	Spearman correlation coefficient(r)
Pain and discomfort	PD	1, 2, 3, 4, 26	1~ 4	5	20	(1 + 2 + 3 + 4 + 26)/5	[(RS-1)/3] × 100	0.881^**^
Eating	ET	5, 6, 7, 8	1~ 4	4	16	(5 + 6 + 7 + 8)/4	[(RS-1)/3] × 100	0.975^**^
Saliva	SA	11, 12	1~ 4	2	8	(11 + 12)/2	[(RS-1)/3] × 100	0.918^**^
Social contact	SC	15, 16, 17, 18, 23	1~ 4	5	20	(15 + 16 + 17 + 18 + 23)/5	[(RS-1)/3] × 100	0.939^**^
Diet	DT	21, 22	1~ 4	2	8	(21 + 22)/2	[(RS-1)/3] × 100	0.923^**^
Teeth	TE	9	1~ 4	1	4	9	[(RS-1)/3] × 100	0.984^**^
Opening mouth	OM	10	1~ 4	1	4	10	[(RS-1)/3] × 100	0.938^**^
Sense of taste	TA	13	1~ 4	1	4	13	[(RS-1)/3] × 100	0.997^**^
Appearance	AP	14	1~ 4	1	4	14	[(RS-1)/3] × 100	0.817^**^
Sexuality	SX	19	1~ 4	1	4	19	[(RS-1)/3] × 100	/ ^a^
Oral ulcer	OU	20	1~ 4	1	4	20	[(RS-1)/3] × 100	0.990^**^
Shoulder & neck function	SN	24	1~ 4	1	4	24	[(RS-1)/3] × 100	0.374^*^
Bleeding Gingiva	BG	25	1~ 4	1	4	25	[(RS-1)/3] × 100	0.997^**^
Weight loss	WL	27	1~ 2	1	2	27	(RS-1) × 100	0.866^**^
Weight gain	WG	28	1~ 2	1	2	28	(RS-1) × 100	/ ^a^

*P < 0.05 **p < 0.01

^a^ Each subject got 0 points in the sexuality scale and weight gain scale in the retest trial (constant sequence), so these scales were not part of the calculation of the correlation coefficient

### Reliability

Cronbach’s α was calculated using data collected during the first trial to confirm internal consistency. In the social contact (α = 0.889) and diet scales (α = 0. 751) satisfying outcomes were achieved (α > 0.7), while internal consistency was slightly lower in the pain and discomfort (α = 0. 677), eating scale (α = 0.515), and saliva scale (α = 0.605). Overall internal consistency was high (α = 0.875).

Data acquired from both trials (with an interval of 24 to 48 h between trials) were used to calculate test-retest reliability; this calculation procedure consisted of comparing the scores from the two trials using correlation coefficient r (Table 3). Except for the shoulder and neck function scales, the tested scales indicated close correlation. Therefore, overall internal consistency was confirmed.

### Validity

Correlation coefficients between the five scales used to establish discriminant validity are shown in Table 4. Diet, eating, and social contact were moderately correlated (0.478–0.551) while pain, saliva, and other scales demonstrated low correlation.

**Table 4:** Correlation coefficient between each scale of QOL-OC

Scales	PD	ET	SA	SC
PD				
ET	.341^**^			
SA	.232^**^	.336^**^		
SC	.154^*^	.539^**^	.283^**^	
DT	.339^**^	.476^*^	.330^**^	.470^**^

Abbreviations: *PD* pain and discomfort, *ET* eating, *SA* saliva, *SC* social contact, *DT* diet

*p < 0.05

**p < 0.01

Correlation coefficients between items and their own scales used to establish convergent validity were calculated (Table 5). Higher correlations were observed between all items and their corresponding scales than between items and the other scales which indicated fairly good convergent validity. There was a lower correlation between the painkiller item and its scale (0.376); however, this value was still greater than the correlations between any other items and the pain and discomfort scale by horizontal or vertical standards for comparison. Finally, the eating fluid item manifested a low correlation (0.372) with the eating scale.

**Table 5:** Spearman correlation coefficient between items and its corresponding scales of QOL-OC

Items		Scales				
		PD	ET	SA	SC	DT
31	Pain in the mouth	**.694** ^**^	.277^**^	.182^**^	.050	.253^**^
32	Pain in the jaw	.525**	.263^**^	.245^**^	.116	.234^**^
33	Pain in the throat	.478**	.200^**^	.199^**^	.218^**^	.201^**^
34	Discomfort in the mouth	.729**	.324^**^	.150^*^	.187^**^	.315^**^
56	Use of painkillers	.376**	.128	.175^**^	.116	.136^*^
35	Eating fluid	.001	.372**	.063	.203^**^	.116
36	Eating semifluid	.245^**^	.506**	.171^*^	.299^**^	.218^**^
37	Eating solid	.322^**^	.905**	.274^**^	.502^**^	.451^**^
38	Choked when eating	.271^**^	.553**	.346^**^	.325^**^	.342^**^
41	Dry mouth	.164^*^	.211^**^	.861**	.200^**^	.268^**^
42	Sticky saliva	.256^**^	.341^**^	.779**	.274^**^	.282^**^
45	Difficult chatting	.043	.392^**^	.183^**^	.809**	.364^**^
46	Social contact with families	.113	.384^**^	.193^**^	.691**	.241^**^
47	Social contact with friends	.136^*^	.422^**^	.229^**^	.789**	.329^**^
48	Social contact in public	.175^**^	.457^**^	.183^**^	.826**	.367^**^
53	Pronunciation	.145^*^	.551^**^	.258^**^	.850**	.457^**^
51	Not enjoying eating	.300^**^	.453^**^	.334^**^	.382^**^	.854**
52	Diet change	.283^**^	.381^**^	.257^**^	.442^**^	.905**

Abbreviations: *PD* pain and discomfort, *ET* eating, *SA* saliva, *SC* social contact, *DT* diet

Correlation coefficients between items and its corresponding scales were made in bold

*p < 0.05

**p < 0.01

Correlation between the C30 and QOL-OC scales was also calculated (Table 6). QL in C30 and ET in OC (*r* = 0.420), SF in C30 and DT in OC (*r* = 0.450) were moderately correlated. There was significant correlation between PA in C30 and PD in OC (*r* = 0.543); significant correlation was also observed between SF in C30 and SC in OC (*r* = 0.525).

**Table 6:** Correlation between scales of C30 and QOL-OC

Scales	QL	PF	RF	EF	CF	SF	FA	NV	PA
PD	−.255^**^	−.192^**^	−.231^**^	−.239^**^	−.271^**^	−.165^*^	.369^**^	.198^**^	**.543** ^**^
ET	**−.420** ^**^	−.327^**^	−.283^**^	−.190^**^	−.069	−.388^**^	.360^**^	.140^*^	.241^**^
SA	−.377^**^	−.182^**^	−.204^**^	−.236^**^	−.214^**^	−.322^**^	.244^**^	.087	.202^**^
SC	−.387^**^	−.277^**^	−.301^**^	−.210^**^	.023	**−.525** ^**^	.278^**^	.049	.079
DT	−.359^**^	−.237^**^	−.344^**^	−.220^**^	−.172^*^	**−.450** ^**^	.323^**^	.103	.293^**^

Abbreviations in QOL-OC: *PD* pain and discomfort, *ET* eating, *SA* saliva, *SC* social contact, *DT* diet

Abbreviations in C30: *QL* global health status, *PF* physical function, *RF* role function, *EF* emotional function, CF cognitive function, *SF* social function, *FA* fatigue, *NV* nausea and vomiting, *PA* pain

Data in bold referred to moderate or higher correlation

*p < 0.05

**p < 0.01

## Discussion

Head and neck cancer is a disease of the upper aerodigestive tract and is one of the most frequently diagnosed cancers worldwide. A high rate of cancers involving the head and neck are reported across the Asian region [44, 45]. To better reflect current clinical management of oral cancer within Chinese, Quality-of-life questionnaire is expected to benefit practitioners when making decisions regarding optimal treatment strategies for their patients.

The Chinese oral cancer specific quality-of-life questionnaire (QOL-OC) categorizes 29 scored questions into 15 scales, five of which are symptom scales and 10 of which are item scales. The QOL-OC also contains a single multi-choice question used for material collection only [27]. In specific clinical application, certain questions can be selectively analysed. But in clinical studies, division of scales can lower the size of data so as to reduce the work load of data analysis.

The telephonic interview was used at this stage and average completion time for the interview was 10.4 min; this was accepted by most of the patients. But it is common for patients refused second interview, one to 2 days after the first one. Asking questions item by item guaranteed that missing data was not an issue in this study. The only missing data concerned sexuality (4.6%), due to a lack of sexual activity among participants of advanced ages [41].

The outcome of the factor analysis coincides basically with our theory. One noteworthy result was that the pain in the throat and discomfort in the mouth items (Items 3 and 4) were placed within the pain and discomfort scale, mainly because of their relevance to clinical functions and the scale division of EORTC-H&N35 [46]. The eating solid and being choked when eating items (Items 7 and 8) were placed within the eating scale together along with the eating fluid and eating semifluid items; this was done in order to guarantee the integrity and logic of this series of questions which concern four levels of difficulty in eating. The sense of taste which was placed within a single scale, was seen as a single item. Weight loss and weight gain are mutually exclusive and opposite, so they were not placed into the same scale. Factor loadings of pain in throat and diet change were high in more than one factors while that of shoulder and neck function and bleeding gingiva were relatively low with all the factors. But these items were not deleted because results may be affected by a small sample size. Nevertheless, the factor analysis demonstrated the statistical significance and clinical value of the items and scale division of the QOL-OC.

The results show that internal consistency reliability was achieved in this research. The reliability was slightly lower in the pain and discomfort and eating and saliva scales, due to statistical correlation and coherence in clinical function. Test-retest reliability for shoulder and neck function was a bit poor; this may be explained by the small size of the sample [38].

Favourable discriminant validity was indicated in that the correlation coefficient was insignificant between scales. This suggests that the different scales measure significantly different symptoms. Convergent validity was demonstrated in that the majority of the items show higher correlation coefficients with their own scales than with the other scales – greater than 0.4 in all cases but one. The painkiller item had a lower correlation (0.378), but this value was still greater than the correlation between the painkiller items and any other scales whether by horizontal or vertical comparison.

It is reported that baseline dysphagia affects multiple domains of QOL and general health perceptions in patients with head and neck cancer prior to treatment. Lango et al. suggested that a dysphagia measure captures the effort of maintaining nutrition, and identifies patients predisposed to disease recurrence and disease-related death [47–49]. Therefore, in our study, according to Chinese people’s eating habits, the eating fluid item (Item 5) was placed within the eating scale in sequence with Items 6, 7, and 8; this decision was made because it is one item in a specific spectrum of eating-related issues which renders its inclusion necessary to achieving a logical outcome. We submit that the eating solid item may be more closely related to masticatory function while the eating fluid or semifluid items may be affected by food leakage arising from defects in the resection of the mandible or maxilla. The pain and social problems scales are present and highly correlated in both the C30 and OC. This indicates that the OC is coherent with a universally recognized measure for evaluation of symptoms. In addition, the OC covers more specific and distinct problems concerning pain in the oral and maxillofacial region and social problems; these are all graded at several levels.

There are also several limitations in our study. First, apart from questions about sexuality, a number of questions in eating scales and this study cannot be answered by nasogastric feeding tube users. To address this, investigators gave answers to these questions based on the patients’ responses. Second, because patients with serious speaking difficulties and patients who spoke more opaque dialects were difficult to understand in the telephonic interviews, these participants were excluded by necessity. Telephonic interview may lead to selection bias, resulting from patients whose language capacity were impaired severely unable to complete the test, which is much more common among advanced oral cancer patients received surgery plus chemotherapy or/and radiotherapy treatment. In addition to an increase of the sample amount, making investigations in multiple methods and raising the ratio of face to face interview will reduce this bias and expand the sample size of test-retest. Third, a lack of investigators lengthened the time window for data collection. Finally, it is possible that patients might have misunderstood the timeframe for some of the questions. For example, the questions about diet change and weight change were meant to reference only the week previous to the interview, but patients tended to describe a long-term change after being sick or receiving treatment.

Although it is in need of further modification and improvement, this questionnaire is sufficiently reliable and valid for evaluating the quality of life of Chinese oral cancer patients. It is a successful preliminary step in the development of quality-of-life measures specific to Chinese patients with oral cancer.

## Conclusion

Based on literature review and clinic evaluation, Oral Cancer Quality-of-life Questionnaire (QOL-OC) were designed and demonstrates fairly good statistical reliability, validity, and feasibility.

## Additional files

Additional file 1: The English version of QOL-OC, this Oral Cancer Quality-of-life Questionnaire were initially designed and used for Chinese Oral Cancer patients, this is an English translation version. (PDF 298 kb)

Additional file 2: Scores of EORTC-C30, this table shows the primary score of patients answering the C30 questionnaire. (PDF 1500 kb)

Additional file 3: Scores of QOL-OC, this table shows the primary score of patients answering the C30 questionnaire include retest. (PDF 1558 kb)

## Abbreviations

CF: Cognitive function
DT: Diet
EF: Emotional function
EORTC QLQ-C30: Quality of life core questionnaire 30 of the European Organisation for Research and Treatment of Cancer
EORTC: European Organization for Research and Treatment of Cancer
ET: Eating
FA: Fatigue
FACT: Functional Assessment of Cancer Therapy
FLIC: Functional Living Index-Cancer
NV: Nausea and vomiting
PA: Pain
PD: Pain and discomfort
PF: Physical function
QL: Global health status
QOL-OC: Oral Cancer Quality-of-life Questionnaire
RF: Role function
SA: Saliva
SC: Social contact
SD: Standard deviation
SF: Social function
UW-QOL: University of Washington Head and Neck Measure
