# Supplementary material for: Development and evaluation of oral Cancer quality-of-life questionnaire (QOL-OC)
Source: BMC Cancer. 2018 May 3;18:523. doi: 10.1186/s12885-018-4378-6 (PMC5934940; doi:10.1186/s12885-018-4378-6)
Supplement: Supplementary file 1 — The English version of QOL-OC, this Oral Cancer Quality-of-life Questionnaire were initially designed and used for Chinese Oral Cancer patients, this is an English translation version. (PDF 298 kb) [file 12885_2018_4378_MOESM1_ESM.pdf]

The English version of QOL-OC

We are interested in something about you and your health over the past seven days,please answer all of the questions yourself by choosing appropriate option that best applied to you. The information you provide will remain strictly confidential. This questionnaire is used in combination with EORTC QLQ-C30, which include the first thirty question. Thank you again for your support and participation.

During the past week	NO	A little	Quite a lot	Very much
1. Have you felt pain in mouth?				
2. Have you felt pain in jaw?				
3. Have you felt pain in throat?				
4. Have you felt sore/aching in mouth?				
5. Have you had any trouble swallowing liquid (e.g. milk, soup) ?				
6. Have you had any trouble swallowing semifluid (e.g. congee) ?				
7. Have you had any trouble swallowing solid food?				
8. Did you choked when swallowing?				
9. Have you had problems with teeth?				
10. Have you had any trouble opening mouth?				
11. Did you have a dry mouth?				
12. Did you have sticky saliva?				
13. Have you had problems with your sense of taste?				
14. Have you felt being bothered by appearance?				
15. Have you had any trouble talking to others?				
16. Have you had any trouble having social contact with family?				
17. Have you had any trouble having social contact with friends?				
18. Have you had any trouble going out public?				
19. Have you felt your sexual life being affected?				
20. Did you have oral ulcers?				
21. Have you had any trouble enjoying meals?				
22. Did you have changes in your eating habits?				

23. Is your pronunciation clear and loud as usual ？＊				
24. Have you had any trouble with your shoulder and neck？				
25. Did you have bleeding gums？	**no**	**occasionally**	**sometimes**	**usually**
26. Did you use painkillers？	**no**	**occasionally**	**sometimes**	**usually**
27. Did you lose weight？	**yes**		**no**	
28. Did you gain weight？	**yes**		**no**	
29. In the past week, which of the following three have the greatest impact on you?	A. pain<br>B. appearance change<br>C. swallowing function change<br>D. masticatory function change<br>E. language function change<br>F. taste change<br>G. Changes of saliva secretion<br>H. Limitation of shoulder and neck function<br>I. Mood<br>J. Diet change<br>K. Other (please write specific items)			

These are all the contents of this questionnaire, and thanks for your participation and support.
