# Supplementary material for: Development and evaluation of oral Cancer quality-of-life questionnaire (QOL-OC)
Source: BMC Cancer. 2018 May 3;18:523. doi: 10.1186/s12885-018-4378-6 (PMC5934940; doi:10.1186/s12885-018-4378-6)
Supplement: Supplementary file 2 — Scores of EORTC-C30, this table shows the primary score of patients answering the C30 questionnaire. (PDF 1500 kb) [file 12885_2018_4378_MOESM2_ESM.pdf]

Number of patients	Score of C30																													
	1	2	3	4	5	6	7	8	9	10	11	12	13	14	15	16	17	18	19	20	21	22	23	24	25	26	27	28	29	30
1	3	4	3	4	2	3	3	1	2	3	3	4	3	1	1	1	1	3	2	1	1	1	1	2	1	2	2	1	5	4
2	1	1	1	1	1	2	1	1	2	1	1	1	1	1	1	1	1	1	2	1	1	1	1	1	2	1	1	1	5	5
3	1	1	1	1	1	1	1	1	1	1	1	1	1	1	1	1	1	1	1	1	1	1	1	1	1	1	1	2	5	6
4	1	1	1	1	1	1	1	1	1	2	1	1	1	1	1	1	1	1	1	1	1	1	2	1	1	1	1	1	6	6
5	1	2	1	1	1	1	1	2	2	1	2	1	1	1	1	1	1	2	1	1	2	2	2	2	1	1	1	2	5	5
6	1	1	1	1	1	1	1	1	1	1	1	1	1	1	1	2	1	1	1	1	1	1	1	1	1	1	1	1	6	5
7	1	4	4	4	1	2	2	1	1	1	1	1	1	2	2	1	1	1	1	1	1	1	1	1	2	1	2	2	6	6
8	2	2	2	3	1	2	2	1	1	3	1	1	1	1	1	1	1	2	1	1	1	1	1	1	1	2	4	2	5	5
9	1	1	1	1	1	1	1	1	2	2	2	2	1	1	1	1	1	2	1	1	2	3	3	2	2	2	1	2	5	6
10	2	2	2	4	1	1	1	1	1	3	1	3	1	1	1	1	1	2	1	1	1	1	1	1	1	1	1	1	6	6
11	1	1	1	1	1	1	1	1	1	1	1	1	1	1	1	1	1	1	1	1	1	1	1	1	2	1	1	2	6	6
12	1	1	1	1	1	1	1	2	2	2	1	2	1	1	1	1	1	2	2	1	2	2	2	2	4	1	1	2	5	4
13	1	2	1	1	1	1	1	1	1	1	1	1	1	1	1	1	1	1	1	1	1	1	1	1	1	1	1	1	5	7
14	1	1	1	1	1	1	1	1	1	1	1	1	1	1	1	1	1	2	1	1	2	2	2	2	1	1	1	1	5	5
15	1	1	1	1	1	1	1	1	1	1	1	1	1	1	1	1	1	1	1	1	1	1	1	1	1	1	1	1	6	6
16	2	2	1	1	1	1	1	1	2	2	3	2	1	1	1	2	1	2	1	1	1	2	1	1	2	1	1	3	5	5
17	2	2	2	1	1	2	1	1	1	2	1	2	2	1	2	1	1	2	1	1	1	1	1	1	1	1	2	1	7	5
18	1	1	1	1	1	1	1	1	1	2	1	2	1	1	1	2	1	2	1	1	2	2	2	1	2	1	1	2	7	7
19	2	2	1	1	1	2	2	2	2	2	1	2	1	1	1	1	1	2	1	1	1	1	1	2	1	1	1	3	7	3
20	2	2	1	1	1	1	2	1	2	1	1	1	1	1	1	2	1	1	2	1	1	1	1	1	1	1	1	1	5	5
21	1	1	1	1	1	1	1	1	1	1	1	1	1	1	1	1	1	1	1	1	1	1	1	1	1	1	1	1	7	7
22	1	1	1	1	1	1	1	1	1	2	1	2	1	1	1	1	1	1	1	1	1	1	1	1	1	1	1	1	7	7
23	1	1	1	1	1	1	1	1	1	2	2	1	1	1	1	1	1	1	1	1	1	1	1	2	1	1	1	1	6	6
24	1	2	1	1	1	2	2	1	2	1	3	1	1	1	1	1	1	1	1	1	1	1	1	2	2	1	2	1	5	5
25	1	1	1	1	1	3	1	1	1	1	1	2	2	1	1	1	1	1	1	1	1	1	2	2	1	2	2	4	5	5
26	1	1	1	3	1	4	3	1	1	2	1	1	1	1	1	1	1	1	1	1	1	1	1	1	1	1	2	2	6	5
27	1	1	1	1	1	1	1	2	1	1	1	1	1	1	1	1	1	1	1	1	1	1	2	1	2	1	1	1	7	7
28	1	1	1	1	1	2	2	1	2	1	2	1	1	1	1	1	1	1	1	1	1	1	1	1	1	1	1	1	6	6
29	1	1	1	1	1	1	1	1	1	1	1	1	1	1	1	1	1	1	1	1	1	1	1	1	1	1	1	2	7	7
30	1	1	1	1	1	1	1	1	1	1	1	1	1	1	1	1	1	1	1	1	1	1	1	1	1	1	1	1	7	7
31	4	4	1	1	1	2	1	1	2	2	1	1	1	1	1	1	1	1	1	1	1	1	1	1	1	2	1	1	5	5
32	1	1	1	1	1	1	1	1	1	1	1	1	1	1	1	1	1	1	1	1	1	1	1	1	1	1	1	1	6	6
33	2	1	1	1	1	1	2	1	1	2	1	1	1	1	1	1	1	1	1	1	1	1	1	1	1	1	1	1	5	5
34	1	2	1	1	1	1	1	1	4	2	4	2	1	1	1	1	1	2	2	1	1	1	1	1	1	1	4	1	4	4
35	1	1	1	1	1	1	1	1	1	1	1	1	1	1	1	1	1	1	1	1	1	1	2	1	1	3	1	3	4	6
36	2	1	1	2	1	1	1	1	1	1	1	1	1	1	1	1	1	2	1	1	1	1	1	1	2	1	1	1	6	6
37	3	3	1	1	1	2	2	2	2	1	1	1	1	1	1	1	1	2	1	1	1	1	1	1	1	1	1	1	6	6
38	2	1	1	1	1	1	1	1	1	1	1	1	1	1	1	1	1	1	1	1	1	1	1	1	2	1	1	2	6	6
39	1	1	1	1	1	1	1	1	1	1	1	1	1	1	1	1	1	1	1	1	1	1	1	1	1	1	3	1	7	7
40	1	2	1	1	1	1	1	1	1	1	1	1	1	1	1	1	1	1	1	1	1	1	1	1	1	1	1	2	6	6
41	2	1	1	1	1	2	2	1	2	2	1	2	1	1	1	1	1	2	1	1	2	2	2	2	2	2	2	2	4	5
42	2	1	1	1	1	1	1	1	1	2	1	2	1	1	1	1	1	2	1	1	1	1	1	2	2	1	1	1	6	6
43	1	1	1	1	1	1	1	1	1	1	1	1	1	1	1	1	1	1	1	1	1	1	1	1	1	1	1	1	7	7
44	1	1	1	2	1	2	1	1	1	1	1	1	1	1	1	1	1	1	1	1	1	1	1	1	2	1	1	1	6	6
45	3	2	1	1	1	1	1	1	1	2	1	2	2	1	1	2	1	1	1	1	1	1	1	1	2	1	1	2	5	5
46	1	1	1	1	1	1	1	1	1	2	2	1	1	1	1	1	1	3	1	1	1	1	1	1	4	1	1	3	7	7
47	2	1	1	1	1	1	3	1	2	1	2	1	1	1	1	1	1	1	1	1	1	1	1	1	2	1	1	1	5	5
48	1	1	1	1	1	2	2	1	1	2	1	4	1	1	1	1	1	1	2	1	1	1	1	1	1	1	1	1	5	5
49	4	2	1	4	1	1	2	1	1	1	1	2	1	1	1	1	1	1	1	1	1	1	1	1	1	4	4	4	3	5
50	2	1	1	1	1	2	1	1	2	2	2	2	1	1	1	1	1	1	1	1	1	1	2	1	1	1	2	1	5	5
51	1	1	1	1	1	2	1	1	2	1	1	2	1	1	1	1	1	1	1	1	1	1	1	1	1	1	2	3	6	6
52	1	1	1	1	1	1	1	2	1	1	1	1	2	1	1	1	1	1	1	1	1	1	1	1	1	1	1	2	7	7
53	1	1	1	1	1	1	1	1	1	1	1	1	1	1	1	1	1	2	1	1	1	1	1	1	2	1	1	2	6	6
54	2	1	1	1	1	1	1	1	1	1	1	1	1	1	1	1	1	1	1	1	1	1	1	1	1	1	1	2	7	7
55	1	1	1	1	1	1	1	1	2	1	1	1	1	1	1	1	2	2	1	1	1	1	1	1	2	1	1	3	6	6
56	1	1	1	1	1	1	1	2	1	2	1	1	1	1	1	1	1	1	1	1	1	1	2	1	1	1	1	2	6	7
57	1	1	1	1	1	2	1	1	1	2	2	2	1	1	1	1	1	2	1	2	1	1	2	1	1	1	1	1	4	4
58	1	1	1	1	1	1	1	1	1	1	1	1	1	1	1	1	1	1	1	1	1	1	1	1	2	1	1	1	6	6
59	1	1	1	1	1	2	2	1	2	2	1	2	1	1	1	1	1	2	1	1	1	1	1	1	1	2	2	3	5	6
60	1	1	1	1	1	1	1	1	1	1	1	1	1	1	1	1	1	1	1	1	1	1	1	1	2	1	1	1	6	6
61	3	2	1	1	1	1	1	1	3	2	1	2	3	1	1	2	1	2	2	1	1	2	1	1	3	2	2	2	4	4
62	1	1	1	1	1	2	2	1	2	2	1	1	1	1	1	1	1	1	2	1	1	1	2	2	1	1	1	1	5	5
63	1	1	1	1	1	1	1	1	1	1	1	1	1	1	1	1	1	1	1	1	1	1	1	1	2	1	1	1	6	6
64	2	3	1	1	1	2	2	1	1	2	3	1	2	1	1	1	1	1	1	1	1	1	1	2	1	1	4	1	7	7
65	1	1	1	1	1	2	1	1	1	1	2	1	1	1	1	1	1	1	1	1	1	1	2	2	1	1	2	1	7	5
66	2	2	1	1	1	1	1	1	1	2	1	2	1	1	1	1	1	2	1	1	1	1	1	1	2	1	2	3	4	1
67	1	1	1	1	1	2	1	1	1	2	1	2	1	1	1	1	1	2	1	1	2	2	2	2	1	2	1	2	5	5
68	1	1	1	1	1	1	1	1	1	2	2	2	1	1	1	3	3	2	1	1	2	2	2	2	2	3	3	3	2	3
69	1	1	1	1	1	1	1	1	1	1	1	1	1	1	1	1	1	1	1	1	1	1	1	1	2	1	1	2	7	7
70	2	1	1	1	1	1	1	1	1	1	1	1	1	1	1	1	1	1	1	1	1	1	1	1	1	2	2	3	5	5
71	2	1	1	1	1	1	1	1	2	3	1	1	1	1	1	1	1	1	1	1	1	1	1	1	1	1	1	1	7	7
72	2	1	1	1	1	1	1	1	2	2	1	1	1	1	1	1	1	1	1	1	1	1	1	1	1	1	1	1	7	7
73	1	1	1	1	1	1	1	1	1	1	1	1	1	1	1	1	1	1	1	1	1	1	1	1	1	1	1	1	7	7
74	1	1	1	1	1	2	1	1	3	2	2	2	3	2	1	1	1	2	3	1	1	1	2	2	1	1	2	1	4	3

75	2	1	1	1	1	2	2	1	1	1	1	1	1	1	1	2	1	2	1	1	1	1	1	1	1	1	2	1	7	7
76	1	1	1	1	1	1	1	1	1	1	1	1	1	1	1	1	1	1	1	1	1	1	1	1	1	1	1	1	7	7
77	3	3	2	1	1	1	1	2	3	2	1	1	1	2	2	2	1	3	1	1	1	1	2	1	2	3	1	2	7	3
78	1	1	1	1	1	1	1	1	1	1	1	1	1	1	1	1	1	1	1	1	1	1	1	1	2	1	1	1	6	5
79	1	1	1	1	1	1	1	1	1	1	1	1	1	1	1	1	1	1	1	1	1	1	1	1	1	1	1	1	7	7
80	1	1	1	1	1	1	1	1	1	1	1	1	1	1	1	1	1	1	1	1	1	1	1	1	2	1	1	2	5	6
81	1	1	1	1	1	1	1	1	2	1	1	1	2	1	1	2	1	1	1	1	1	1	2	1	2	1	2	1	5	5
82	1	1	1	1	1	1	1	1	3	2	2	2	1	1	1	1	1	2	1	1	1	1	1	1	2	2	2	2	4	3
83	1	1	1	1	1	1	1	1	2	1	1	1	1	1	1	1	2	1	1	1	2	2	3	2	3	2	1	2	5	5
84	1	4	1	1	1	2	2	2	1	2	1	2	1	1	1	2	1	2	1	1	1	1	3	1	3	1	1	2	3	4
85	1	1	1	1	1	1	1	1	1	2	1	2	1	1	1	2	1	1	1	1	1	1	2	1	1	1	3	1	6	6
86	2	2	1	1	1	1	1	1	2	2	1	2	2	1	1	1	1	2	2	1	1	2	1	2	3	1	1	2	7	7
87	1	1	1	1	1	1	1	1	1	2	1	1	1	1	1	1	1	1	1	1	1	1	1	1	1	1	1	1	7	7
88	2	2	1	1	1	1	1	1	2	1	1	1	1	2	1	1	1	1	1	2	1	1	2	1	1	1	2	3	4	5
89	2	1	1	1	1	2	1	1	2	2	4	3	1	1	1	1	2	2	2	1	2	2	1	2	2	1	1	1	4	4
90	1	1	1	1	1	1	1	1	1	1	1	1	1	1	1	1	1	1	1	1	1	1	1	1	1	1	3	3	7	6
91	1	1	1	1	1	1	1	1	1	1	1	1	1	1	1	1	1	1	1	1	1	1	1	1	2	2	2	2	6	6
92	2	1	1	1	1	1	1	1	2	3	1	1	1	1	1	1	1	1	2	1	1	1	1	1	1	1	1	1	7	7
93	1	1	1	1	1	1	1	1	1	1	1	1	1	1	1	1	1	1	1	1	1	1	1	1	2	1	1	1	6	6
94	1	1	1	1	1	1	1	1	1	1	1	1	1	1	1	1	1	1	1	1	1	1	1	1	1	4	4	4	3	2
95	4	2	1	2	3	3	2	1	1	2	2	2	1	1	1	1	1	1	1	1	1	1	3	1	1	2	3	3	4	2
96	3	3	3	3	3	3	1	2	1	2	3	1	1	1	1	1	1	1	1	1	1	1	2	1	1	3	1	3	5	5
97	3	3	3	1	1	1	1	1	1	2	1	2	1	1	1	2	1	2	1	1	1	1	1	1	1	3	3	4	5	3
98	1	1	1	1	1	1	1	1	1	1	1	1	1	1	1	1	1	1	1	1	1	1	1	1	1	1	1	2	6	7
99	1	1	1	1	1	1	1	1	1	2	1	1	1	1	1	1	1	1	1	1	1	1	1	1	1	1	1	2	7	7
100	2	1	1	1	1	1	1	2	2	4	1	4	1	1	1	2	1	4	1	1	2	2	2	2	4	1	1	1	4	4
101	3	1	1	1	1	1	1	1	1	2	2	1	1	1	1	1	1	1	1	1	1	1	1	1	2	1	1	3	5	4
102	2	1	1	1	1	1	1	1	1	1	1	1	1	1	1	1	1	1	1	1	1	1	1	1	1	1	1	3	7	7
103	1	1	1	1	1	1	1	1	1	1	1	1	1	1	1	2	1	1	1	1	1	1	2	1	1	1	1	1	5	5
104	3	1	1	1	1	2	2	1	2	2	2	2	1	1	1	1	1	2	2	2	1	1	1	1	2	2	2	3	5	5
105	3	1	1	1	1	1	1	1	2	1	1	2	2	2	1	2	2	2	1	1	1	1	1	1	3	1	1	2	7	6
106	1	1	1	1	1	2	2	1	1	1	1	1	2	1	1	1	1	1	1	1	1	1	1	1	2	2	2	2	6	7
107	2	2	1	2	1	2	1	1	2	2	2	1	1	1	1	1	1	2	2	2	1	1	2	1	1	1	2	2	5	5
108	3	3	3	1	1	2	1	1	1	1	3	2	2	1	1	2	1	2	1	1	1	1	2	2	2	1	2	2	5	5
109	1	1	1	1	1	1	1	1	2	1	1	1	2	1	1	2	1	1	1	1	2	1	1	1	1	2	1	1	4	4
110	1	1	1	1	1	1	1	1	1	1	1	1	1	1	1	1	1	1	1	1	1	1	1	1	2	2	2	1	6	6
111	1	1	1	1	1	1	1	1	1	1	1	1	1	1	1	1	1	1	1	1	1	1	1	1	1	1	1	2	5	5
112	2	2	1	1	1	2	1	1	3	2	1	2	1	1	1	3	1	2	2	1	1	1	1	1	1	1	1	1	5	6
113	2	3	2	1	1	2	1	2	2	1	2	2	1	1	1	1	1	2	2	1	1	1	2	1	2	2	2	3	4	5
114	1	1	1	1	1	1	1	1	1	2	1	1	1	1	1	1	1	1	1	1	1	1	1	1	1	1	2	1	7	7
115	4	4	3	3	1	3	3	2	3	3	3	2	1	1	1	1	1	2	3	2	1	1	1	1	2	3	4	4	4	4
116	2	1	1	1	1	1	2	1	1	2	2	1	1	1	1	1	1	1	1	1	2	2	1	1	2	1	2	1	6	5
117	1	1	1	1	1	1	1	1	1	2	1	1	1	1	1	1	1	2	1	1	1	1	1	1	1	1	1	1	4	4
118	2	3	2	3	1	1	1	1	1	3	4	1	2	1	1	1	1	1	3	1	2	1	1	1	4	1	1	2	5	5
119	2	2	1	1	1	1	1	2	2	1	1	1	1	1	1	1	1	1	1	1	2	2	2	2	1	2	1	2	5	3
120	1	1	1	1	1	1	1	1	4	3	4	2	1	1	1	1	1	2	3	1	1	1	2	3	2	2	1	3	1	1
121	1	1	1	1	1	1	1	1	1	1	1	1	1	1	1	1	1	1	1	1	1	1	1	1	2	1	1	2	6	6
122	1	1	1	1	1	2	2	1	1	1	1	1	1	1	1	1	1	1	1	1	1	1	1	1	1	2	2	1	5	5
123	1	1	1	1	1	2	1	1	1	1	2	1	1	1	1	1	1	1	1	1	1	1	1	1	2	3	3	2	5	1
124	2	1	1	1	2	3	3	1	2	2	2	2	1	1	1	1	1	1	2	1	1	1	1	1	2	1	3	1	5	5
125	2	1	1	1	1	1	1	1	3	1	2	1	2	1	1	1	1	2	2	1	1	1	2	1	1	3	3	3	7	7
126	1	1	1	1	1	1	1	1	1	1	1	1	1	1	1	2	1	1	1	2	2	2	1	2	2	1	1	1	6	6
127	3	2	1	1	1	1	1	1	2	2	1	2	2	1	1	2	1	2	2	1	1	1	1	1	2	1	1	1	4	5
128	1	1	1	1	1	1	1	1	3	2	1	2	1	1	1	2	1	2	1	1	1	1	1	1	1	1	1	1	5	5
129	1	3	1	3	2	2	3	1	1	2	1	2	1	1	1	2	1	2	1	1	1	2	2	2	1	3	3	2	4	4
130	1	1	1	1	1	1	1	1	2	2	2	2	3	1	1	1	1	2	1	2	1	2	2	2	1	1	1	2	5	5
131	3	1	1	2	1	1	1	1	2	2	2	2	1	1	1	1	1	2	1	1	1	1	1	1	1	2	1	2	6	6
132	1	1	1	1	1	1	1	1	1	1	1	1	1	1	1	1	1	1	1	1	1	1	1	1	1	1	1	1	6	6
133	1	1	1	1	1	1	1	1	2	1	1	2	1	1	1	1	1	1	1	1	1	1	1	1	1	1	1	3	6	5
134	1	1	1	1	1	1	1	1	1	1	1	1	1	1	1	1	1	1	1	1	1	1	1	1	1	1	2	3	7	7
135	1	1	1	1	1	3	3	1	2	2	1	1	1	1	1	1	1	1	1	1	1	1	1	1	1	2	3	3	6	6
136	1	1	1	1	1	1	2	1	1	2	1	2	1	1	1	2	1	2	1	1	1	1	1	1	1	1	1	2	6	7
137	1	1	1	1	1	1	1	1	1	1	2	1	1	1	1	1	1	2	1	1	1	1	1	1	1	1	1	3	5	5
138	1	1	1	1	1	1	1	1	1	1	1	1	1	1	1	2	1	1	1	1	1	1	1	1	2	1	1	2	5	5
139	1	1	1	1	1	1	1	1	1	1	2	1	1	1	1	2	1	1	1	1	1	1	3	1	2	1	1	1	3	3
140	2	1	1	1	1	1	2	2	2	2	3	1	1	1	1	2	1	2	1	1	2	2	4	2	2	2	1	2	5	4
141	2	2	1	1	1	1	1	2	2	2	2	2	1	1	1	1	1	2	1	1	1	1	3	2	3	1	1	1	5	5
142	1	2	1	1	1	1	1	1	1	2	1	1	2	1	1	2	1	1	1	1	1	1	2	1	2	1	1	2	5	5
143	2	2	1	1	1	1	1	1	1	1	1	1	1	1	1	1	1	1	1	1	1	1	1	1	2	1	1	2	5	5
144	2	1	1	1	1	2	2	1	2	1	1	1	2	1	1	1	1	1	2	1	1	1	2	1	1	2	2	2	5	5
145	1	1	1	1	1	1	1	1	1	1	2	1	1	1	1	1	1	1	1	1	1	1	1	1	1	1	1	1	6	6
146	2	2	1	1	1	2	2	1	1	1	1	1	1	1	1	1	1	1	1	1	1	1	2	1	1	2	2	3	3	2
147	2	1	1	1	1	1	1	1	1	1	1	1	1	1	1	1	1	1	1	1	1	1	2	1	2	1	2	2	6	6
148	1	1	1	1	1	1	1	1	1	1	1	1	1	1	1	2	1	1	1	1	1	2	2	1	2	1	2	1	5	5
149	2	2	1	1	1	2	1	2	2	1	2	2	1	1	1	1	2	2	2	1	1	2	1	2	1	2	2	3	5	5
150	1	1	1	1	1	1	1	2	1	1	2	2	2	1	1	1	1	2	1	1	1	2	2	1	1	3	2	3	5	5

151	1	2	1	1	1	1	1	1	1	1	1	1	1	1	1	1	1	1	1	1	1	1	1	1	1	1	1	4	4	4
152	2	2	1	1	1	1	1	1	1	2	1	1	1	1	1	1	1	2	1	1	1	1	1	1	1	1	1	3	5	5
153	1	1	1	1	1	1	1	1	1	1	1	1	1	1	1	1	1	1	1	1	1	1	1	1	1	1	1	2	6	6
154	2	2	1	1	1	1	1	2	3	2	2	2	2	1	1	1	1	2	2	1	2	2	2	2	1	2	2	2	4	4
155	2	3	2	2	2	3	3	1	2	2	1	2	3	1	1	1	1	2	1	1	1	1	1	2	2	3	2	3	3	3
156	2	2	1	1	1	2	1	1	2	1	1	1	1	1	1	1	1	1	1	1	1	1	2	1	1	1	2	1	5	4
157	1	1	1	1	1	1	1	1	2	1	1	2	1	1	1	1	1	1	1	1	1	2	1	1	2	1	1	1	5	5
158	2	2	1	1	1	1	1	1	1	1	1	2	1	1	1	1	1	1	1	1	1	1	2	1	2	2	2	2	5	5
159	1	1	1	1	1	1	1	1	1	1	1	1	1	1	1	1	1	1	1	1	1	1	1	1	1	1	2	1	6	6
160	1	1	1	1	1	1	1	1	1	1	1	1	1	1	1	1	1	1	1	1	1	1	1	1	1	1	1	2	7	7
161	1	1	1	1	1	1	1	1	2	1	1	1	1	1	1	1	1	1	1	1	1	1	1	1	1	1	1	1	5	5
162	1	2	1	1	1	1	1	2	1	1	1	1	1	1	1	1	2	1	1	1	1	1	1	1	1	1	1	2	7	7
163	2	1	1	1	1	2	1	1	1	2	1	1	1	1	1	1	1	1	1	1	1	1	1	1	1	1	1	1	5	5
164	1	1	1	1	1	2	1	1	1	1	1	2	1	1	1	1	1	2	1	1	1	1	2	2	2	1	3	3	5	5
165	1	2	1	1	1	4	4	1	1	1	1	1	1	1	1	1	1	1	1	1	1	1	1	1	1	1	1	1	7	7
166	1	1	1	1	1	1	1	1	1	1	3	1	1	1	1	1	1	1	1	1	1	1	1	1	1	1	1	1	5	6
167	1	2	1	1	1	1	2	1	1	1	1	1	1	1	1	1	1	1	1	1	1	1	1	1	1	1	1	2	6	6
168	3	1	1	1	1	1	1	1	1	1	3	1	3	1	1	1	1	1	1	1	1	1	2	1	1	1	1	3	5	5
169	1	1	1	2	1	1	1	1	2	2	3	2	2	2	2	1	1	1	2	1	1	1	1	1	2	2	1	3	5	5
170	2	1	1	1	1	1	1	1	1	1	1	1	1	1	1	1	1	1	1	1	1	1	1	1	1	1	1	1	6	6
171	2	1	1	1	1	1	1	1	2	2	1	1	1	1	1	1	1	1	1	1	1	1	1	1	1	1	3	1	7	7
172	1	2	1	1	1	1	1	1	1	1	2	1	1	1	1	1	1	1	1	1	1	1	1	1	1	2	1	1	7	7
173	1	1	1	1	1	1	1	1	2	1	3	2	1	1	1	2	1	1	1	1	2	2	3	2	3	1	1	3	6	6
174	1	1	1	1	1	1	2	1	1	1	1	1	1	1	1	1	1	1	1	1	1	1	1	1	2	1	1	1	7	7
175	3	1	1	1	1	1	2	1	3	2	2	1	1	1	1	1	1	1	3	1	1	1	1	1	2	1	1	2	5	5
176	1	1	1	1	1	1	1	1	1	2	3	1	1	1	1	1	1	1	1	1	1	1	1	1	1	1	1	1	7	7
177	1	1	1	1	1	1	1	2	1	1	2	1	1	1	1	1	1	1	1	1	1	1	1	1	1	1	1	1	7	7
178	1	1	1	1	1	1	1	1	1	1	1	1	1	1	1	1	1	1	1	1	1	1	1	1	1	1	1	1	6	6
179	1	1	1	1	1	2	1	1	2	2	1	1	1	1	1	1	1	2	1	2	2	2	2	1	3	1	1	2	6	6
180	3	3	2	2	3	3	3	1	3	1	2	1	3	1	1	1	1	1	2	1	2	2	2	2	1	1	1	3	4	4
181	1	2	1	1	1	1	1	1	1	2	1	1	1	1	1	1	1	2	1	1	1	1	2	1	1	1	1	1	7	7
182	4	4	1	3	1	2	1	1	1	1	1	1	1	1	1	1	1	2	1	1	1	1	1	1	1	1	1	2	6	6
183	1	1	1	1	1	1	1	1	1	1	3	1	1	1	1	1	1	1	1	1	1	1	1	1	1	1	1	2	6	6
184	3	3	1	1	1	2	1	1	1	2	4	2	1	1	1	1	1	2	1	1	1	1	1	1	2	2	2	2	5	6
185	2	3	2	1	1	2	2	1	1	2	1	1	3	1	1	1	1	2	1	1	1	1	1	1	1	3	3	2	6	6
186	1	2	1	1	1	1	2	1	1	1	1	1	1	1	1	1	1	1	1	1	1	1	1	1	1	1	1	3	6	6
187	2	2	1	1	1	1	1	1	1	1	2	1	1	1	1	1	1	1	1	1	1	1	1	1	2	1	2	1	6	6
188	1	1	1	1	1	1	1	1	1	1	1	1	1	1	1	1	1	1	1	1	1	1	1	1	1	1	1	1	6	6
189	1	1	1	1	1	1	1	2	1	1	2	1	1	1	1	1	1	1	1	1	1	1	1	1	1	1	1	1	5	5
190	1	1	1	1	1	1	1	1	1	1	1	1	1	1	1	1	1	1	1	1	1	1	1	1	1	1	1	1	7	7
191	1	1	1	1	1	1	1	1	1	1	1	1	1	1	1	1	1	1	1	1	1	1	1	1	1	1	2	1	6	6
192	2	2	1	1	1	2	1	1	1	2	2	2	2	1	1	1	1	2	1	1	1	1	1	1	2	1	1	1	5	5
193	3	3	1	2	1	3	3	1	2	2	4	3	2	1	1	2	1	2	1	1	2	2	2	2	2	3	3	1	5	5
194	2	2	1	1	1	1	1	1	2	1	1	1	1	1	1	1	1	1	2	1	1	1	1	1	1	1	1	1	7	7
195	1	2	1	1	1	1	1	2	1	1	1	1	1	1	1	1	1	1	1	1	1	1	1	1	1	1	1	3	7	7
196	1	2	1	1	1	1	1	1	2	1	1	2	1	1	1	1	1	1	1	1	2	2	1	2	1	2	2	2	4	5
197	1	2	1	1	1	1	1	1	1	1	1	1	1	1	1	1	1	1	1	1	1	1	1	1	1	1	1	3	7	6
198	1	1	1	1	1	1	1	2	2	2	1	1	3	1	1	2	1	2	2	1	1	3	2	3	2	1	2	1	4	4
199	2	1	1	1	1	1	1	1	1	1	1	1	1	1	1	1	1	1	1	1	1	1	2	1	2	1	1	2	6	6
200	1	1	1	1	1	1	1	1	2	1	1	1	1	1	1	1	1	1	1	1	1	1	1	1	1	1	1	1	7	7
201	1	1	1	1	4	3	3	1	3	4	1	3	2	1	1	3	1	3	3	1	2	2	2	2	1	4	4	3	4	4
202	1	1	1	1	1	1	1	1	1	1	1	1	1	1	1	1	1	1	1	1	1	1	1	1	1	1	1	2	6	6
203	1	1	1	1	1	1	1	1	1	1	2	1	1	1	1	1	1	1	1	1	1	1	1	1	1	1	1	1	5	7
204	1	1	1	1	1	1	1	1	2	1	1	1	1	1	1	1	1	1	1	1	1	1	1	1	1	1	1	1	7	7
205	3	3	2	3	3	3	3	1	2	3	3	3	3	1	1	1	1	3	3	2	1	1	1	1	2	2	3	2	3	3
206	1	1	1	1	1	1	1	1	2	3	1	1	1	1	1	1	1	1	2	1	1	1	1	1	1	1	1	1	6	7
207	2	2	1	1	1	1	3	1	3	2	1	2	2	1	1	2	1	1	2	1	2	2	2	1	1	2	1	2	1	1
208	3	3	1	4	3	3	1	1	2	3	3	2	1	1	1	3	1	1	2	1	1	1	1	2	1	1	1	2	5	6
209	2	2	1	3	2	3	3	1	1	2	2	2	1	1	1	1	1	2	1	2	1	1	1	2	1	2	2	3	6	6
210	1	1	1	1	1	1	1	1	1	1	2	1	1	1	1	1	1	1	1	1	1	1	1	1	2	1	1	2	6	6
211	1	1	1	1	1	2	1	1	2	1	2	2	2	1	1	1	1	1	1	1	2	1	1	1	2	1	1	2	6	7
212	3	3	2	1	1	3	3	1	2	1	2	2	1	2	2	1	1	1	1	1	1	1	1	1	2	2	2	3	5	6
213	3	2	1	2	1	2	1	1	1	1	1	1	1	1	1	1	1	1	1	1	1	1	2	1	1	1	1	1	7	7
