# Supplementary material for: Development and evaluation of oral Cancer quality-of-life questionnaire (QOL-OC)
Source: BMC Cancer. 2018 May 3;18:523. doi: 10.1186/s12885-018-4378-6 (PMC5934940; doi:10.1186/s12885-018-4378-6)
Supplement: Supplementary file 3 — Scores of QOL-OC, this table shows the primary score of patients answering the C30 questionnaire include retest. (PDF 1558 kb) [file 12885_2018_4378_MOESM3_ESM.pdf]

Score of OC and retest

Number of patients	31	32	33	34	35	36	37	38	39	40	41	42	43	44	45	46	47	48	49	50	51	52	53	54	55	56	57	58	59
1	2	1	1	2	1	2	4	2	4	1	2	1	1	2	1	1	1	2	1	1	2	1	2	3	1	1	1	1	CDH
2	2	2	1	2	1	1	2	1	1	2	2	1	1	1	1	1	1	1	1	1	2	2	1	1	1	1	1	1	AGJ
3	1	1	1	1	1	1	1	1	1	1	2	2	1	1	2	1	1	1	1	1	2	3	2	1	1	1	1	1	EGJ
4	1	1	1	1	3	3	3	1	1	1	2	2	1	1	4	1	1	1	缺失	1	1	1	4	1	1	1	1	1	E
5	2	1	1	1	1	1	3	1	3	2	1	2	4	2	1	1	1	1	2	1	2	1	1	2	1	1	1	1	K nerve ache
6	1	1	1	1	1	1	2	2	1	1	2	2	1	1	1	1	1	1	1	1	1	1	1	1	1	1	2	1	C
7	1	1	1	3	1	1	2	1	1	1	1	1	1	1	2	2	2	2	4	1	1	1	3	2	1	1	2	1	E
8	1	1	1	1	1	1	1	2	1	1	2	2	1	1	1	2	2	2	4	1	1	1	1	1	1	1	1	2	K social
9	3	1	1	1	1	1	1	1	1	1	2	2	2	1	1	1	1	1	2	1	1	1	1	1	1	1	1	2	AFI
10	1	1	1	1	1	1	4	1	4	1	2	1	1	1	2	1	1	1	1	1	1	1	3	1	1	1	1	1	DE
11	1	1	1	1	1	1	1	1	1	1	1	1	1	2	1	1	1	1	1	1	1	1	1	1	1	1	1	2	无
12	1	1	1	1	1	1	1	1	1	2	1	1	1	2	1	1	1	1	1	1	1	1	2	2	1	1	1	1	无
13	1	1	1	1	1	1	1	1	1	1	2	2	1	1	1	1	1	1	1	1	1	1	1	2	1	1	1	1	H
14	1	1	1	1	1	1	1	1	2	1	1	1	1	1	1	1	1	1	1	1	1	1	1	1	1	1	1	1	无
15	1	1	1	1	1	1	2	1	2	1	1	1	1	1	1	1	1	1	缺失	1	1	1	1	1	1	1	1	1	无
16	2	1	1	2	1	1	4	2	4	1	2	1	1	1	1	1	1	1	缺失	1	2	2	1	2	1	1	1	1	DK sleep
17	1	1	1	1	1	1	3	3	1	2	1	2	1	1	2	1	2	2	1	1	2	3	3	2	1	1	2	1	CDJ
18	1	1	1	1	1	1	1	1	1	1	1	1	1	1	1	1	1	1	1	1	2	2	2	1	1	1	1	1	BDJ
19	1	1	1	1	1	1	3	1	2	2	1	2	1	1	2	1	2	2	2	1	1	3	4	1	1	1	1	2	DEJ
20	2	1	1	2	1	1	1	1	1	1	4	4	1	1	1	1	2	2	1	1	1	2	2	1	1	1	1	2	EG
21	1	1	1	1	1	1	2	1	1	1	1	1	1	1	1	1	1	1	1	1	1	1	1	1	1	1	1	1	无
22	1	1	1	3	1	1	3	1	1	2	1	1	3	1	1	1	1	1	1	1	1	2	2	1	1	1	1	1	DIJ
23	1	1	1	1	1	1	1	1	1	1	2	1	2	1	1	1	1	1	1	1	1	1	2	1	1	1	1	1	EFG
24	2	1	1	2	1	1	4	1	2	2	1	1	2	4	1	1	1	2	1	1	3	3	2	1	1	1	1	1	BDJ
25	1	3	3	3	1	1	4	2	2	1	3	3	4	3	1	1	1	1	4	2	3	3	3	4	3	1	1	1	BGH
26	1	1	1	1	1	1	2	2	1	2	4	1	1	1	2	2	2	2	3	1	3	3	2	1	1	1	1	1	DGJ
27	1	1	1	1	1	1	1	1	1	1	1	2	2	1	2	1	1	1	1	1	1	3	4	2	2	1	1	1	CK tongue
28	2	1	1	1	1	1	1	1	1	1	2	1	1	2	1	1	1	1	1	1	2	3	1	1	1	1	1	1	ABJ
29	2	1	1	1	1	1	1	1	1	1	1	1	1	1	2	1	1	2	1	1	1	1	1	2	1	1	1	1	EH
30	1	1	1	1	1	1	1	1	1	1	1	1	1	1	1	1	1	1	1	1	1	2	1	1	1	1	1	1	J
31	2	1	1	2	1	1	2	1	1	1	1	1	1	1	2	1	2	2	1	1	3	3	2	1	1	1	2	1	ADJ
32	1	1	1	1	1	1	1	1	1	1	1	1	2	2	1	1	1	1	1	1	1	1	2	1	2	1	1	1	F
33	1	1	1	1	1	1	1	1	1	1	2	1	1	1	1	1	1	1	1	1	2	2	2	1	1	1	1	2	C
34	4	4	1	4	1	4	4	2	3	2	4	3	1	1	4	3	3	4	1	1	4	4	4	3	1	4	1	2	AEG
35	1	1	1	1	1	1	1	1	1	2	1	2	1	1	2	1	1	2	1	1	2	2	2	1	1	1	1	2	EGJ
36	1	1	1	1	1	1	1	1	1	1	1	1	1	1	1	1	1	1	1	1	1	2	1	1	1	1	1	2	EJK social
37	1	1	1	2	1	1	2	1	1	2	2	2	1	1	1	1	1	1	1	2	2	1	1	3	2	1	1	1	CDH
38	2	1	1	1	1	1	2	2	1	1	2	2	2	1	1	1	1	1	1	2	2	1	1	1	1	1	1	2	CFG
39	1	1	1	1	1	1	1	1	1	1	2	2	1	2	2	1	2	2	2	1	1	1	2	1	1	2	1	2	BGH
40	1	1	1	1	2	1	1	1	1	2	1	1	1	1	1	1	1	1	1	1	1	1	1	1	1	1	1	1	DF
41	1	1	2	2	1	1	1	1	1	1	2	1	1	1	2	1	2	2	1	1	2	2	2	1	1	2	2	1	EJ
42	2	1	1	2	1	1	4	1	1	2	1	1	1	2	2	1	2	2	1	1	2	2	2	1	1	1	1	1	DIJ
43	1	1	1	2	1	1	1	1	1	1	1	2	1	1	1	1	1	1	1	1	2	1	2	2	1	1	1	2	HJ
44	1	1	1	1	2	1	1	3	1	1	1	1	1	2	1	1	1	1	1	1	3	2	2	1	1	1	1	1	CJ
45	2	2	1	2	1	1	4	1	2	3	1	1	1	2	1	1	1	1	1	1	2	2	1	1	1	1	1	1	D
46	2	1	1	2	1	1	1	1	2	1	1	1	1	2	1	1	1	1	1	1	1	1	1	2	1	1	1	1	B
47	1	1	1	2	1	1	2	1	1	1	1	2	1	3	1	1	1	1	1	1	1	1	1	2	1	1	1	1	BDG
48	2	2	1	2	1	1	3	1	1	2	1	1	1	2	2	1	1	2	2	1	1	1	2	2	1	1	1	1	AGH
49	1	1	1	1	1	1	3	3	1	1	1	2	4	3	3	2	2	3	4	1	4	4	4	2	1	1	1	1	BCF
50	1	1	2	1	1	1	2	1	2	1	2	2	1	1	2	1	1	2	1	2	1	1	2	2	1	1	1	1	CEH
51	1	1	1	1	1	1	4	2	2	1	2	1	2	3	3	2	3	3	1	1	2	2	3	2	1	1	1	2	CHJ
52	2	1	1	1	1	1	1	1	2	1	1	1	1	1	1	1	1	1	1	1	1	2	1	1	1	1	1	2	J
53	1	1	1	2	1	1	4	1	2	1	1	2	1	2	1	1	1	1	1	1	1	1	1	2	1	1	1	1	BDH
54	1	1	1	1	1	1	1	1	2	2	2	2	1	1	1	1	1	2	1	1	1	1	1	1	1	1	1	2	K open mouth
55	1	1	1	2	1	1	1	1	2	1	1	1	1	1	1	1	1	1	1	1	2	2	1	1	1	1	1	1	J
56	1	1	1	1	1	1	2	2	2	1	2	1	1	1	1	1	1	1	1	1	1	1	2	1	1	1	1	1	CDG
57	1	1	1	2	1	1	2	1	2	2	2	2	1	2	1	1	1	1	1	1	1	1	1	1	1	1	1	1	CDJ
58	1	1	1	1	1	1	4	1	1	1	2	1	1	1	1	1	1	1	1	1	2	1	2	1	1	1	1	1	DE
59	2	1	2	2	1	1	2	1	1	2	2	1	1	1	2	2	2	2	1	2	3	3	2	2	1	1	1	1	EIJ
60	1	1	1	1	1	1	1	1	1	1	3	1	1	1	1	1	1	1	1	1	1	1	1	1	1	1	1	1	G
61	3	3	3	1	1	1	1	2	3	1	3	3	3	1	3	2	2	1	3	1	3	1	3	3	2	3	2	1	AFJ
62	2	2	1	2	1	1	4	2	2	3	3	3	2	2	2	2	2	2	1	1	4	3	4	1	1	1	1	2	DEJ
63	1	1	1	1	1	1	2	1	1	1	1	1	1	1	1	1	1	1	1	1	1	1	1	1	1	1	1	2	
64	1	2	1	2	2	4	4	1	2	2	1	2	1	3	2	2	2	3	1	1	1	3	3	2	2	1	1	2	BEJ
65	1	1	1	1	2	1	2	1	1	2	1	2	1	2	2	1	2	2	1	1	1	3	3	1	1	1	1	1	DEJ
66	1	1	1	1	3	3	3	1	2	2	1	2	2	1	3	3	4	4	2	2	2	3	3	1	1	1	1	1	BEG
67	1	1	1	2	2	1	2	2	2	2	2	1	1	2	2	1	2	2	1	2	1	2	3	1	1	1	1	2	BEK
68	1	1	1	1	1	1	4	1	2	1	3	3	1	2	3	3	3	3	1	3	1	1	3	1	2	1	1	1	CEG
69	1	1	1	1	1	1	2	1	3	2	1	1	1	1	1	1	1	1	1	1	2	2	1	2	1	1	1	2	CD
70	1	1	1	1	1	1	2	1	2	1	1	1	1	1	2	1	1	2	1	1	1	1	2	2	1	1	1	2	
71	1	2	1	1	1	1	1	1	2	2	3	3	1	1	1	1	1	1	1	1	1	1	1	1	2	1	1	1	
72	2	1	1	2	1	1	1	1	1	2	2	1	1	1	1	1	1	1	1	1	2	1	1	1	1	1	1	2	
73	1	1	1	1	1	1	1	1	1	1	1	1	1	2	1	1	1	1	1	1	1	1	1	1	1	1	1	1	
74	3	2	3	2	1	1	3	2	1	1	4	1	4	1	1	1	1	1	1	2	3	3	3	1	1	1	2	1	EJ
75	1	1	1	1	1	1	1	1	1	2	2	2	1	2	1	1	1	1	1	1	1	1	1	1	1	1	1	1	
76	1	1	1	1	2	2	1	1	1	1	2	1	1	1	1	1	1	1	1	1	1	1	1	1	2	1	1	1	G
77	4	4	1	3	1	1	3	2	2	2	4	4	2	4	1	1	1	1	3	2	2	3	2	2	1	1	2	1	CDJ
78	1	1	1	1	1	1	2	1	2	1	1	1	1	1	1	1	1	1	1	1	2	2	1	1	1	1	1	1	
79	1	1	1	1	1	1	1	1	1	1	1	1	1	1	1	1	1	1	1	1	1	1	1	1	1	1	1	2	K none
80	1	1	1	2	1	1	3	1	1	3	2	2	1	2	2	1	2	2	1	3	2	2	2	1	2	1	1	1	CDE
81	1	2	1	2	1	1	3	1	3	2	2	1	1	1	1	1	1	2	1	2	2	3	2	1	1	1	1	1	DEJ

82	2	2	1	2	1	1	4	3	1	4	1	4	2	2	1	2	2	2	2	1	2	2	3	4	1	4	1	1	BCE
83	2	1	1	2	1	1	3	2	3	2	3	2	1	1	1	1	1	1	2	1	1	2	3	3	3	1	1	2	ADE
84	1	1	2	2	1	1	2	2	2	3	2	3	1	3	1	1	2	2	1	1	1	2	1	1	1	1	2	1	BDE
85	1	2	1	2	1	1	3	1	1	2	2	1	1	2	1	1	1	2	1	1	1	2	2	2	1	1	1	2	GH
86	1	1	1	2	1	1	2	1	2	1	2	1	2	1	1	1	1	1	1	1	1	2	1	1	1	1	1	1	GIK
87	1	1	1	1	1	1	1	1	1	1	2	1	1	1	1	1	1	1	1	1	1	1	1	1	1	1	1	2	DGJ
88	1	1	1	2	1	1	2	1	1	2	2	2	2	1	1	1	1	1	1	1	1	2	2	2	1	1	1	1	BH
89	2	1	1	1	1	1	2	1	1	1	1	1	1	1	1	1	1	1	1	1	1	1	1	3	1	1	1	2	AHK
90	1	1	1	2	1	1	4	1	1	1	2	2	1	2	1	1	1	2	1	1	3	3	3	1	1	1	1	1	BDE
91	1	1	1	1	1	1	1	1	1	1	4	2	1	1	2	1	1	1	2	1	1	1	1	1	1	1	1	1	
92	2	1	1	1	2	2	1	1	2	1	1	2	1	1	1	1	1	1	1	1	1	2	1	1	1	1	2	1	
93	1	1	1	1	1	1	1	1	2	1	2	3	1	1	2	1	2	1	2	1	1	1	2	1	1	1	1	2	EG chin
94	1	1	1	1	3	3	3	1	4	2	2	1	1	1	3	3	3	3	1	1	3	1	4	1	1	1	1	1	DE
95	1	1	1	1	3	3	3	1	1	1	2	4	1	3	2	1	3	3	1	1	3	1	3	1	1	1	2	1	
96	2	1	1	1	1	2	3	1	1	1	2	1	1	1	2	2	2	2	1	1	2	1	3	1	1	1	2	1	
97	1	1	1	1	1	1	4	2	1	2	3	2	1	2	3	1	1	1	4	1	2	3	3	2	1	1	1	1	E walk
98	1	1	1	1	1	1	1	1	1	1	1	1	1	4	1	1	1	1	1	1	1	1	2	1	1	1	1	1	B
99	1	1	1	1	1	1	4	1	3	1	2	2	1	1	1	1	1	1	缺失	1	1	2	2	1	1	1	1	1	
100	2	2	2	2	4	4	1	2	2	2	3	4	1	2	3	3	3	3	4	1	4	3	3	1	1	1	2	1	BEG every
101	1	1	1	1	1	1	1	2	2	1	3	2	1	1	1	1	1	1	2	1	1	1	1	2	1	1	1	1	GTinnitus and insomnia
102	2	1	1	2	1	1	1	1	1	1	1	1	1	1	1	1	1	1	1	1	1	1	1	1	1	1	1	1	gum pain
103	1	1	2	1	1	1	1	1	1	2	2	1	1	2	1	1	1	1	缺失	1	1	2	1	1	1	1	1	1	BI
104	2	1	1	1	1	1	1	1	2	1	3	1	1	3	1	1	1	1	缺失	1	2	2	1	1	1	1	1	1	I tired
105	2	2	2	2	1	1	1	2	3	2	1	1	2	1	1	1	2	2	1	2	2	2	1	1	2	1	1	1	D
106	1	2	1	2	1	1	1	1	2	2	2	2	2	1	1	1	1	1	1	1	1	2	1	1	2	1	1	1	
107	3	2	2	3	2	3	3	1	3	2	2	2	1	2	1	1	1	2	缺失	1	1	3	4	1	1	1	1	1	AC
108	1	1	2	1	1	1	1	1	2	2	2	1	1	1	1	1	1	2	1	1	1	1	4	1	1	1	2	1	
109	2	1	1	1	1	1	3	1	1	1	2	1	1	1	2	1	1	2	1	1	1	1	1	1	1	1	1	1	AD
110	1	1	1	1	1	1	1	1	1	1	2	1	1	1	1	1	1	1	1	1	1	2	1	2	2	1	1	2	Less sedentary and activity in the evening
111	1	1	1	1	1	1	2	1	2	2	2	1	1	1	1	1	1	1	1	1	1	1	1	2	1	1	1	1	
112	3	1	1	2	1	1	3	1	3	2	1	1	1	2	2	1	1	2	1	2	2	3	2	1	1	1	1	2	ADE
113	2	1	1	1	1	1	2	2	1	1	2	2	2	2	2	2	2	2	1	1	1	2	3	2	1	1	1	1	CDE
114	1	1	1	2	1	1	1	1	2	1	1	1	1	1	1	1	1	2	1	1	1	3	2	1	1	1	1	1	CDE
115	1	2	2	2	2	2	4	3	2	1	1	2	1	2	3	3	3	3	1	1	4	3	4	1	1	4	1	1	ADE
116	1	1	1	1	1	1	1	1	2	1	2	1	1	2	1	1	1	1	1	1	4	4	2	1	1	1	1	1	BEJ
117	1	1	1	1	1	1	1	1	2	1	1	1	1	1	1	1	1	1	1	1	1	1	2	2	1	1	1	1	tired
118	2	1	1	3	1	2	4	1	3	3	3	2	1	1	1	1	1	4	1	1	4	1	2	2	1	1	1	2	GH sleep
119	1	1	1	1	1	1	1	1	1	1	2	2	1	1	1	1	1	1	1	1	1	1	1	1	2	1	1	1	A
120	3	1	1	1	1	1	3	2	1	1	3	1	1	1	1	1	1	1	1	1	2	2	1	2	1	2	2	1	A
121	2	1	1	1	1	1	1	1	1	1	2	1	1	1	1	1	1	1	1	1	1	1	1	2	1	4	1	1	AGH
122	1	1	1	2	1	1	1	1	1	1	1	1	1	1	1	1	1	1	1	2	1	2	2	1	2	1	1	1	lichen planus
123	1	1	1	2	1	1	4	1	1	1	2	1	2	2	2	2	2	2	1	2	2	2	3	1	2	1	1	2	EJ Dry throat
124	2	1	1	2	1	1	3	2	2	2	2	2	2	2	3	1	1	1	1	2	4	3	3	3	1	1	2	1	EJCan’t smoke and drink
125	1	1	1	1	1	1	2	1	1	1	2	1	2	2	1	1	1	1	1	2	2	2	2	2	1	1	1	1	Can’t smoke and drink，social
126	2	1	1	1	1	1	1	1	1	1	2	1	1	2	1	1	1	1	2	1	2	1	1	1	1	1	1	2	无
127	2	1	1	1	1	1	2	1	2	2	3	1	1	1	1	1	1	1	缺失	1	2	2	2	1	1	1	1	2	DK sleep
128	2	1	1	1	1	1	1	1	1	1	1	1	1	2	1	1	1	1	1	2	1	2	2	1	1	1	1	1	sleeo
129	1	1	1	1	1	1	3	1	2	1	2	2	4	2	2	1	1	1	2	3	4	3	2	2	1	3	2	1	EF
130	2	2	2	2	1	1	2	1	1	2	2	2	2	1	1	1	1	1	缺失	1	2	1	3	1	1	1	1	2	A
131	1	1	1	1	1	1	1	1	2	1	1	1	1	1	1	1	1	1	缺失	1	2	1	2	3	1	1	1	1	A
132	1	1	1	2	1	1	1	1	1	2	1	1	1	1	2	1	1	1	1	1	2	2	2	2	1	1	1	2	E
133	1	1	1	2	1	1	4	1	1	1	1	3	1	3	1	2	1	1	1	1	1	2	2	1	1	1	1	2	H
134	1	1	1	2	1	1	1	1	2	2	2	1	1	2	1	1	1	1	2	1	1	3	2	2	4	1	1	1	AH
135	1	1	1	2	1	1	4	1	1	2	1	3	2	3	1	1	2	3	1	1	2	3	2	3	1	1	1	1	DHJ
136	1	1	1	1	1	1	1	1	1	1	2	2	1	1	2	1	1	2	1	1	2	3	2	1	1	1	1	2	J
137	1	1	1	2	1	1	2	1	2	1	2	2	1	1	1	1	1	1	1	1	1	1	2	1	1	1	1	2	DE
138	1	1	1	1	1	1	1	1	1	1	1	1	1	1	1	1	1	1	1	1	1	1	1	1	1	1	1	2	
139	1	1	1	2	1	1	2	2	2	2	2	2	1	3	2	1	2	2	1	3	3	3	2	1	1	1	2	1	BDE
140	1	1	1	1	1	1	1	1	1	1	3	3	2	1	2	2	2	2	1	1	4	4	1	2	1	1	1	2	FG
141	2	2	1	1	1	1	1	1	1	2	3	2	2	1	1	1	1	1	1	1	1	2	1	2	1	1	2	1	BF
142	1	1	1	1	1	1	2	1	2	1	4	1	2	1	2	2	2	2	1	1	2	2	2	1	1	1	2	1	DEF
143	1	1	1	1	1	1	3	1	2	1	4	1	1	1	2	2	2	2	1	1	1	1	2	2	1	1	1	2	FG swollen tongue
144	2	1	1	2	1	1	3	2	1	1	3	3	3	2	2	2	2	2	1	1	2	3	3	2	1	1	1	2	DEG swollen tongue
145	1	1	1	1	1	1	1	1	2	1	2	1	1	1	2	2	2	2	1	2	1	1	2	1	1	1	1	2	
146	1	1	1	1	1	1	3	1	3	1	2	2	1	1	2	2	2	2	1	1	2	3	2	2	1	1	2	1	EJ numbness
147	1	1	1	2	1	1	2	1	2	1	2	1	2	1	1	1	1	1	1	1	2	2	3	2	1	1	1	1	EJ
148	1	1	2	2	1	1	3	1	3	2	2	2	1	1	2	1	1	2	1	1	2	2	3	2	1	1	1	2	EJ
149	1	1	1	1	1	1	2	1	2	1	1	1	1	1	2	2	2	2	1	1	2	2	2	2	1	1	2	1	DE
150	1	1	1	1	1	1	3	3	1	1	2	2	2	2	2	2	2	2	1	1	2	2	3	2	1	1	1	1	EIJ
151	1	1	1	1	1	1	2	1	2	2	1	1	1	1	2	2	2	2	1	1	1	1	2	1	1	1	1	2	DE swollen tongue
152	1	1	1	2	1	1	3	1	3	1	1	1	1	1	2	2	2	2	1	1	1	2	3	2	1	1	1	2	DEJ
153	1	1	1	1	1	1	1	1	2	1	1	1	1	1	1	1	1	1	1	1	2	2	2	1	1	1	1	2	D
154	3	1	1	3	1	1	3	2	2	3	3	1	1	2	2	2	2	2	1	1	3	3	2	1	2	1	2	1	AG
155	2	1	1	2	1	2	3	2	1	3	2	2	3	1	1	1	1	1	1	1	2	3	3	3	1	1	2	1	DEH
156	1	1	1	1	1	1	1	1	1	2	1	1	1	1	1	1	1	1	1	1	1	1	1	2	1	1	1	2	
157	1	1	1	1	2	1	1	1	1	1	2	1	2	1	1	1	1	1	1	1	1	1	2	3	1	1	1	1	
158	1	1	1	2	1	1	2	1	2	3	2	2	1	1	1	1	1	1	1	1	2	2	2	2	1	1	1	1	EGJ
159	1	1	1	1	1	1	1	1	1	1	2	1	1	3	2	2	2	2	1	1	1	1	3	1	1	1	1	1	be
160	1	1	1	2	1	1	2	1	2	2	1	1	1	1	2	2	2	2	1	1	1	1	2	1	1	1	1	1	ce
161	1	1	1	2	2	2	3	1	2	3	2	2	1	1	1	1	1	1	1	2	3	3	3	2	1	1	1	1	a
162	1	1	1	2	1	1	1	1	1	1	1	1	1	2	2	2	2	2	1	1	1	1	3	1	1	1	1	1	g
163	1	1	1	2	1	1	1	1	1	1	2	3	1	1	1	1	1	1	2	1	2	2	2	2	1	1	1	1	gh
164	1	1	1	1	1	1	4	2	1	1	4	2	4	2	2	1	1	2	1	1	2	3	3	1	1	1	2	1	dgj

165	1	1	1	1	1	1	1	1	2	3	3	1	1	2	1	1	1	1	2	2	1	1	2	2	1	1	1	1	b
166	1	1	1	2	1	1	1	1	3	1	2	1	1	1	1	1	1	1	1	1	1	1	1	1	1	1	1	1	
167	1	1	1	2	1	1	2	1	1	1	1	1	1	2	1	1	1	1	1	1	1	1	2	1	1	1	1	1	
168	3	1	2	2	1	2	4	1	2	2	4	3	4	3	2	2	2	2	1	3	3	4	3	2	1	1	2	1	fgj
169	1	2	2	2	1	1	4	1	2	2	2	3	1	1	1	1	1	1	1	1	2	3	2	1	1	1	2	1	a
170	1	1	1	1	1	1	2	1	1	1	1	1	1	1	1	1	1	1	1	2	1	1	2	1	1	1	1	1	e
171	1	1	1	1	1	1	3	2	1	1	1	1	1	3	3	3	3	3	1	1	1	2	3	2	1	1	1	2	bde
172	1	1	1	2	1	1	3	2	3	3	4	2	3	3	1	1	1	1	1	1	2	1	2	1	1	1	1	1	bdg
173	1	1	1	2	1	1	1	1	1	1	1	1	1	1	1	1	1	1	1	2	1	1	1	2	1	1	1	1	i
174	1	1	1	1	1	1	1	1	1	1	4	4	4	1	1	1	1	1	1	1	1	3	2	1	1	1	1	1	fg
175	3	1	2	2	1	1	2	1	2	1	2	3	1	2	1	1	1	1	1	1	2	1	1	1	1	2	2	1	
176	1	1	1	2	1	1	1	1	1	1	1	1	1	1	1	1	1	1	1	1	1	1	1	1	1	1	1	1	
177	1	2	1	1	1	1	1	1	1	1	1	2	1	1	1	1	1	1	1	1	1	1	1	1	1	1	1	1	
178	1	1	1	1	1	1	1	1	2	1	1	1	1	1	1	1	1	1	1	1	1	2	1	1	1	1	1	2	
179	1	1	1	2	1	1	1	1	2	2	1	1	2	1	2	1	1	1	1	1	2	3	1	1	1	1	1	1	
180	1	2	1	1	1	1	2	1	2	1	1	2	1	1	1	1	1	1	1	1	1	1	1	1	1	1	1	1	a
181	1	1	1	2	1	1	1	1	2	1	1	1	1	1	1	1	1	1	1	1	1	1	1	1	1	1	1	1	
182	1	1	1	1	1	1	1	1	1	1	2	1	1	2	1	1	1	1	1	1	1	1	1	1	1	1	1	1	B
183	1	1	1	2	1	1	2	1	1	2	1	1	1	1	2	2	2	1	1	1	1	2	1	2	1	1	1	2	DHJ
184	1	1	1	3	1	1	4	1	3	3	4	4	2	1	3	2	3	2	1	2	3	4	2	3	1	1	2	1	DGH
185	1	1	1	1	1	1	4	2	2	1	2	1	1	4	2	2	2	2	1	1	3	3	2	3	1	1	2	1	BCH
186	1	1	1	1	1	1	4	1	1	1	2	1	1	3	2	1	2	2	1	1	3	3	3	1	1	1	1	2	BDE
187	1	1	1	1	1	1	2	1	1	1	2	1	2	2	1	1	1	1	1	1	3	2	2	1	1	1	1	2	BJ
188	1	1	1	2	1	1	3	1	1	1	2	1	1	2	1	1	1	1	1	1	3	2	1	1	1	1	2	1	
189	1	1	1	1	1	1	1	1	2	1	2	2	1	1	1	1	1	1	1	1	1	1	1	1	1	1	1	2	
190	1	1	1	1	1	1	1	1	1	1	1	1	1	1	1	1	1	1	1	1	1	1	1	2	1	1	1	1	
191	1	1	1	1	1	1	2	1	2	1	1	1	1	2	1	1	1	2	1	2	1	1	1	2	1	1	1	2	H
192	1	1	1	2	1	1	2	1	1	1	2	2	1	2	1	1	2	2	1	2	2	2	1	2	1	1	1	1	BEH
193	1	1	1	1	1	1	1	1	1	2	3	1	1	2	2	1	1	2	1	1	1	3	2	2	4	1	2	1	BDG
194	1	2	1	1	1	1	1	1	2	1	1	1	1	2	1	1	1	1	1	2	1	2	1	1	1	1	1	1	A
195	1	1	1	1	1	1	2	1	1	2	1	1	1	2	1	1	1	1	1	1	1	1	2	1	1	1	1	2	
196	1	1	2	1	1	1	1	1	2	2	1	1	1	2	2	1	1	2	1	2	3	3	2	1	1	1	1	1	AJ
197	1	1	1	1	1	1	1	1	1	1	2	1	1	1	2	1	1	1	1	1	1	1	1	1	1	1	1	2	K经济
198	2	2	2	2	1	1	4	2	3	2	3	3	3	3	3	2	3	3	1	2	3	3	3	2	2	1	1	1	DEF
199	1	1	1	2	1	1	1	1	2	1	2	1	1	1	2	2	2	2	1	1	1	1	2	1	1	1	1	2	
200	2	1	1	1	1	1	1	1	1	2	1	1	1	1	1	1	1	1	2	1	1	1	1	1	4	1	1	1	
201	4	1	4	1	1	4	4	1	4	3	1	1	4	1	4	2	2	4	1	1	1	4	4	1	4	2	1	1	BCE
202	1	1	1	1	1	1	1	1	1	1	1	1	1	1	1	1	1	1	1	1	1	1	2	1	1	1	1	1	
203	1	1	2	1	1	1	1	1	1	1	1	1	1	1	1	1	1	1	1	1	1	2	2	1	1	1	1	1	
204	2	1	1	1	1	1	1	1	1	1	1	1	1	1	1	1	1	1	1	1	1	1	1	1	1	1	2	1	
205	3	3	3	1	3	3	3	3	2	2	3	2	1	2	3	3	3	3	1	1	2	3	3	3	1	1	2	1	ADJ
206	2	1	2	2	1	1	1	2	1	1	2	2	1	1	1	1	1	1	1	1	1	1	1	1	1	2	1	1	CJ
207	2	4	1	2	1	2	3	2	3	2	3	2	2	3	2	2	2	2	1	1	3	3	2	1	1	3	2	1	ADE
208	2	1	2	2	2	2	3	2	2	2	1	2	1	1	1	1	1	1	1	1	1	2	2	1	1	1	2	1	AC
209	2	1	2	2	1	1	3	2	1	2	2	2	1	1	2	2	2	2	1	1	3	3	3	1	1	1	2	1	CDE
210	1	1	1	2	1	1	1	1	1	1	1	1	1	1	1	1	1	1	1	1	1	1	1	1	1	1	1	1	
211	2	2	1	1	1	1	1	2	1	1	2	1	1	1	1	1	1	1	1	1	1	1	1	2	1	1	1	1	A
212	2	1	2	2	1	3	3	1	1	2	2	2	1	1	1	1	1	1	1	1	2	1	2	1	1	1	2	1	A
213	1	1	2	1	1	2	3	1	1	2	1	1	1	1	1	1	1	1	1	2	1	1	2	1	1	1	2	1	
200	2	1	1	1	1	1	1	1	1	2	1	1	1	1	1	1	1	1	1	2	1	1	2	1	4	1	1	1	
201	3	1	3	3	1	4	4	1	2	3	1	2	2	2	4	3	3	4	1	1	1	4	4	1	3	3	2	1	ABE
202	1	1	1	1	1	1	1	1	1	1	1	1	1	1	1	1	1	1	1	1	1	1	2	1	1	1	1	1	
203	1	1	2	1	1	1	1	1	1	1	1	1	1	1	1	1	1	1	1	1	1	2	2	1	1	1	1	1	
204	2	1	1	1	1	1	1	1	1	1	1	1	1	1	1	1	1	1	1	1	1	1	1	1	1	1	2	1	
205	3	2	2	2	3	3	3	3	3	3	3	3	1	2	3	3	3	3	1	1	2	3	3	1	1	1	2	1	
206	2	1	1	1	1	1	1	1	1	1	1	2	1	1	1	1	1	1	1	1	1	1	1	1	1	1	1	1	CJ
207	2	4	1	2	1	2	3	2	3	3	3	3	2	3	2	2	2	2	1	1	3	3	2	1	1	3	2	1	
208	2	1	2	2	2	2	3	2	2	2	1	2	1	1	1	1	1	1	1	1	1	2	2	1	1	1	2	1	
209	2	1	2	2	1	1	3	2	1	2	2	2	1	1	2	2	2	2	1	1	1	3	2	2	1	1	2	1	CDE
210	1	1	1	2	1	1	1	1	1	1	1	1	1	1	1	1	1	1	1	1	1	1	1	1	1	1	1	1	
211	2	2	1	1	1	1	1	2	1	1	2	2	1	1	1	1	1	1	1	1	1	1	1	2	1	1	1	1	
212	2	2	1	2	1	3	3	1	1	2	2	2	1	1	1	1	1	1	1	1	2	1	2	1	1	1	2	1	
213	1	1	1	1	1	2	3	1	1	2	1	1	1	1	1	1	1	2	1	1	2	1	2	1	1	1	2	1	EJ
